# A systematic review of the effectiveness of community interventions to improve parent health literacy

**DOI:** 10.1093/eurpub/ckac130.143

**Published:** 2022-10-25

**Authors:** S Belfrage, M Husted, S Fraser, S Patel, J Faulkner

**Affiliations:** Faculty of Health & Well-being, University of Winchester, Winchester, UK; Primary Care, Population Sciences and Medical Education, University of Southampton, Southampton, UK; Department of Paediatric Infectious Diseases, Southampton Children's Hospital, Southampton, UK; Faculty of Health & Well-being, University of Winchester, Winchester, UK; Faculty of Health & Well-being, University of Winchester, Winchester, UK

## Abstract

**Background:**

Many community-based interventions have been developed to increase parent/caregiver health literacy, yet no systematic review of their effectiveness has been published. Therefore, the aim of this systematic review was to examine the effectiveness of community-based health literacy interventions in improving the health literacy of parents/caregivers.

**Methods:**

A systematic review of six databases; MEDLINE, PsycINFO, CINAHL, Cochrane Library, Embase, and Education Source were conducted to identify relevant articles. Risk of bias were assessed using version two of the Cochrane risk of bias tool for randomised controlled trials or the Cochrane Collaboration Risk of Bias in Non-Randomised Studies of Interventions. The study findings were grouped and synthesised following the Synthesis Without Meta-analysis framework.

**Results:**

Eleven community-based health literacy interventions for parents/caregivers were identified. Study design included randomised controlled trials (n = 4), non-randomised studies with comparison group (n = 4), and non-randomised studies without a comparison group (n = 3). Interventions were delivered digitally, in person or a combination of the two. The main findings of the studies showed some potential for both in person and digital interventions to increase parental health literacy. The risk of bias was high in over half the studies (n = 7) Studies were heterogeneous preventing a meta-analysis.

**Discussion:**

Although no definitive conclusion of the effectiveness of community-based interventions can be drawn there are suggestions of improvement in many of the studies included in this review. The review has brought into question whether the health literacy measurement tools used met the needs of assessing the interventions outcomes. When comparing the cost and resources needed for digital with in person interventions, the findings of this review have implications for both practise and research.

**Key messages:**

- Methodologically stronger primary research, informed by theory, is needed to capture the components of effective health literacy intervention for parents.
- Researchers in the field of health literacy need to consider the suitability of using screening tools to measure change in health literacy post intervention.

